# Supplementary material for: Genomic insights into multidrug-resistance, mating and virulence in Candida auris and related emerging species
Source: Nat Commun. 2018 Dec 17;9:5346. doi: 10.1038/s41467-018-07779-6 (PMC6297351; doi:10.1038/s41467-018-07779-6)
Supplement: Supplementary file 1 — Supplementary Information [file 41467_2018_7779_MOESM1_ESM.pdf]

Genomic insights into multidrug-resistance, mating, and virulence in *Candida auris* and related emerging species

José F. Muñoz[1], Lalitha Gade[2], Nancy A. Chow[2], Vladimir N. Loparev[3], Phalasy Juieng[3], Elizabeth L. Berkow[2], Rhys A. Farrer[1], Anastasia P. Litvintseva[2]*, Christina A. Cuomo[1]*

[1] Broad Institute of MIT and Harvard, Cambridge, MA USA. [2] Mycotic Diseases Branch, Centers for Disease Control and Prevention, Atlanta, GA USA. [3] Biotechnology Core Facility Branch, Centers for Disease Control and Prevention, Atlanta, GA USA.

*Corresponding authors: Christina A. Cuomo cuomo@broadinstitute.org; Anastasia P. Litvintseva, frq8@cdc.gov

SUPPLEMENTARY FIGURES

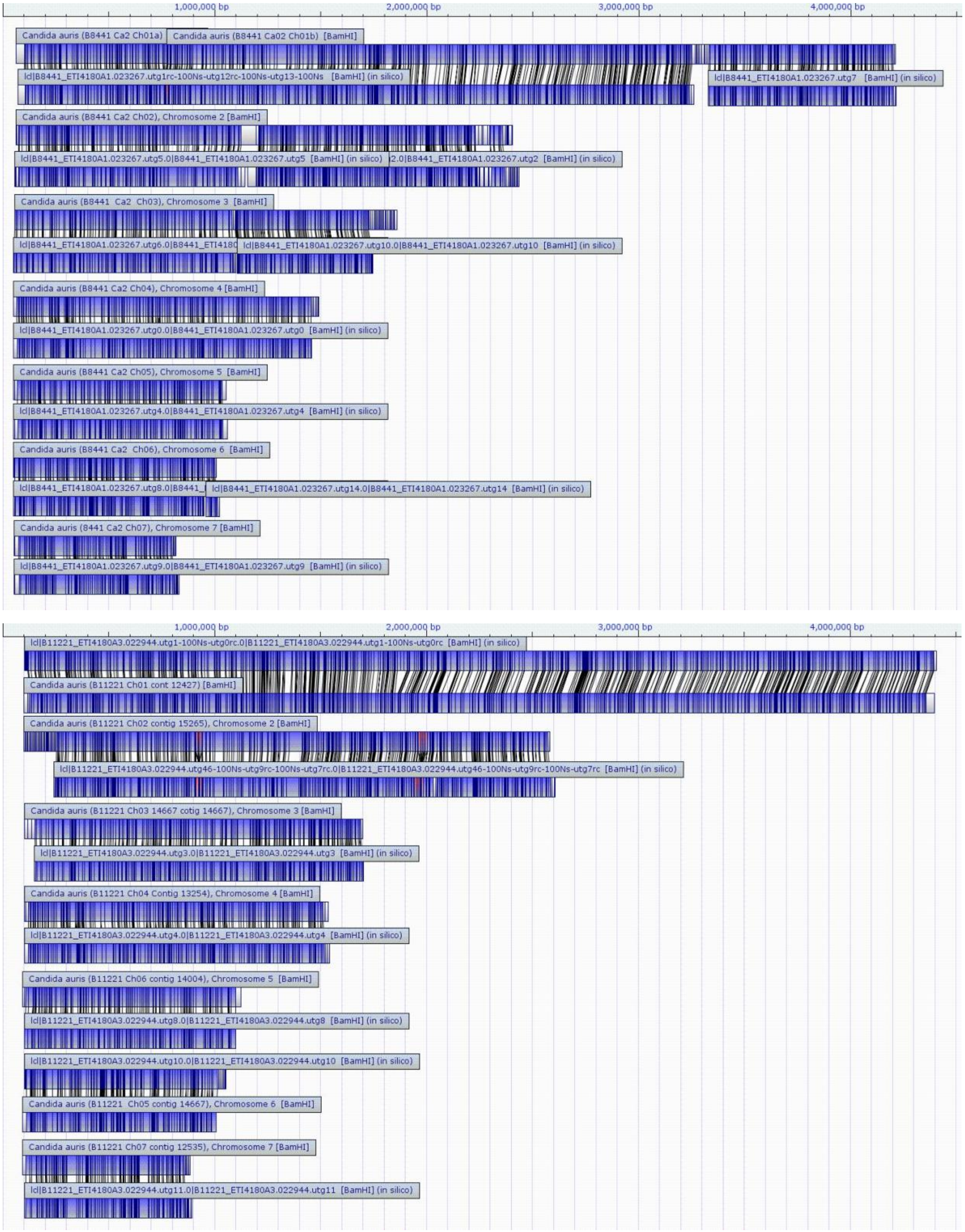

Supplementary Figure 1. Optical map of *Candida auris* strains B8441 (top) and B11221 (bottom).

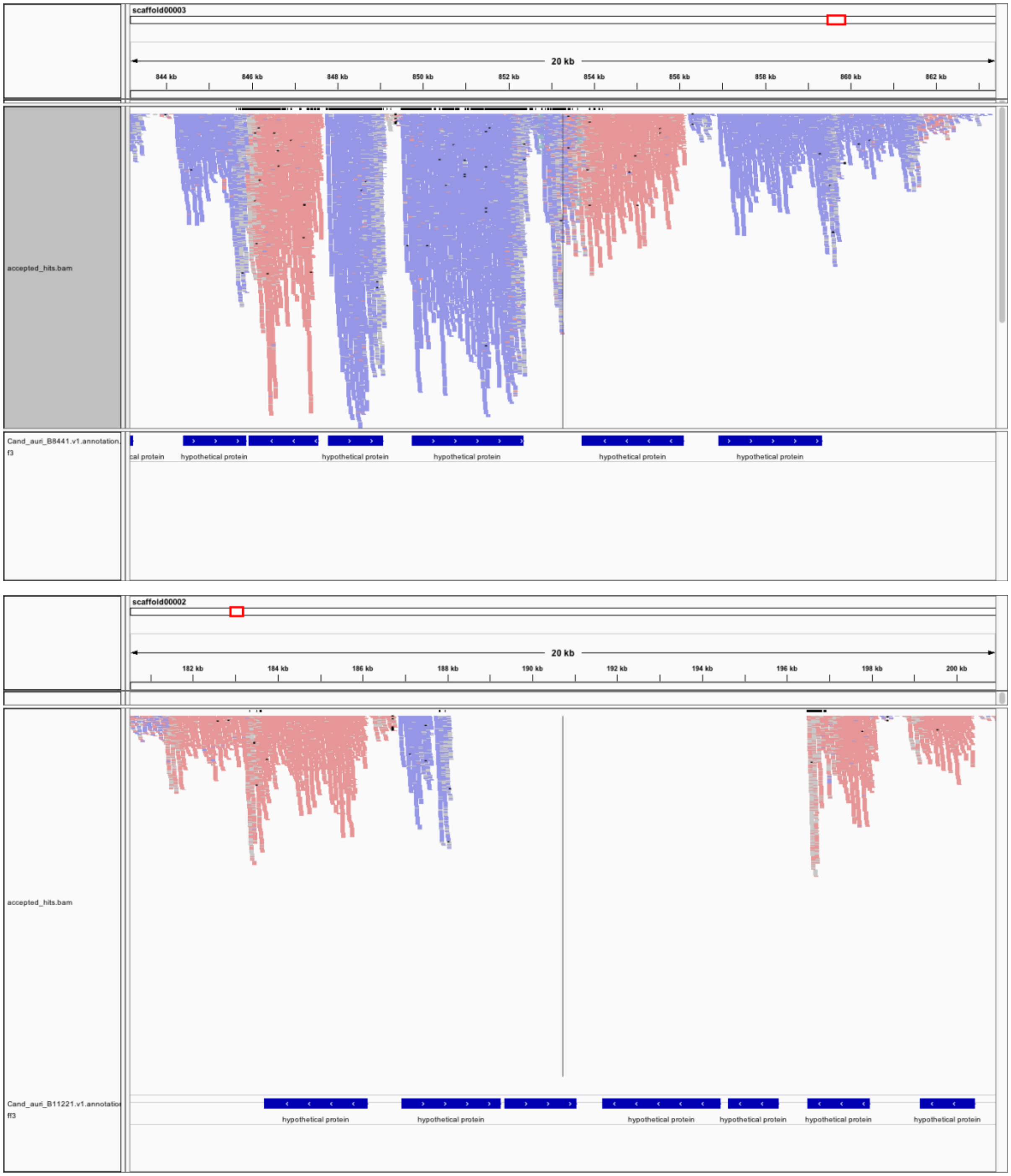

Supplementary Figure 2. RNA-Seq data mapping to the mating type locus. Integrative Genomics Viewer (IGV) snapshots of RNA-Seq Illumina data from *C. auris* strain B8441 (clade I; *MTL****a***) aligned to the annotated genome assembly of *C. auris* B8441 *MTL***a** (top) or to the annotated genome assembly of *C. auris* strain B11221 *MTL*α (bottom).

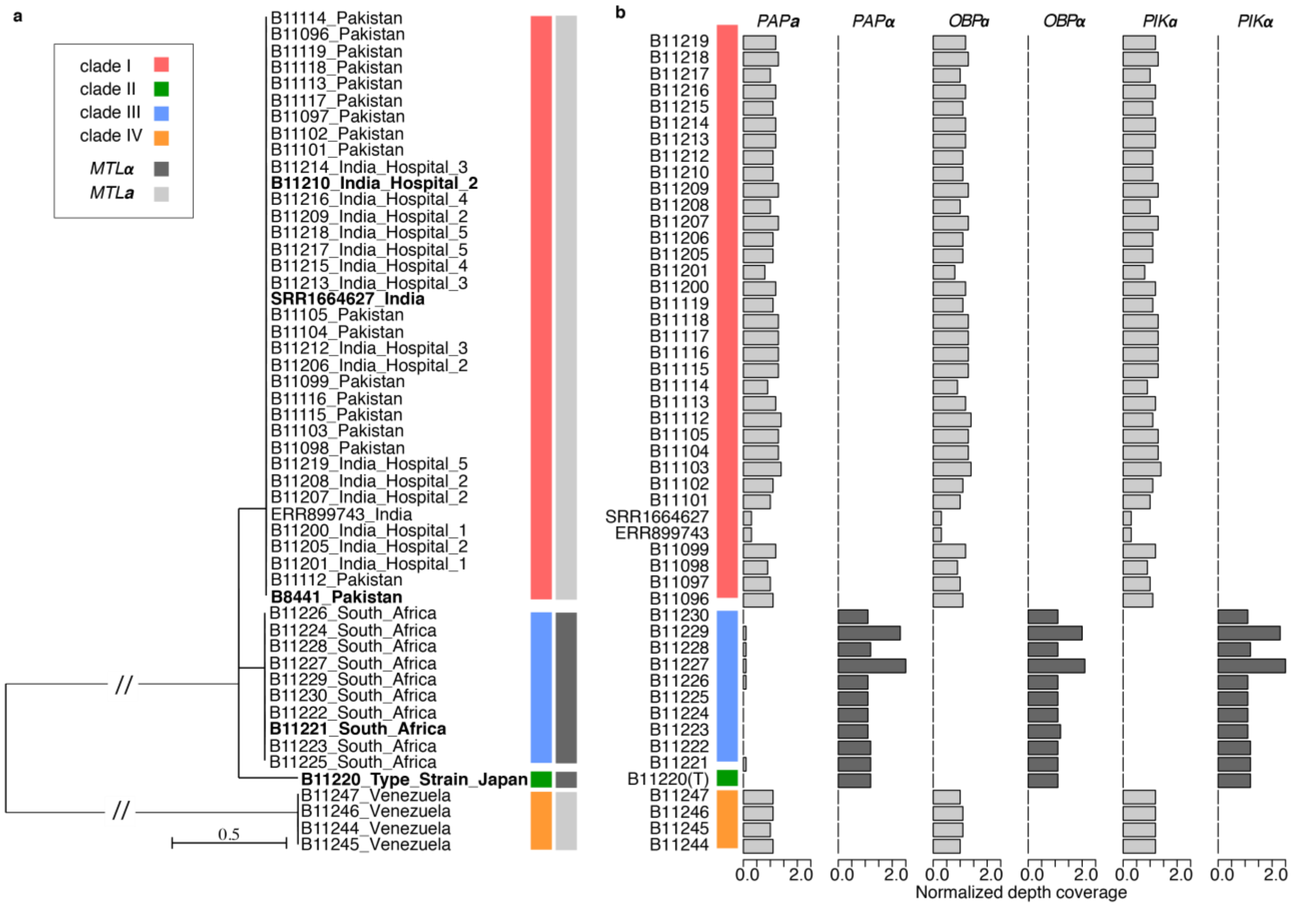

Supplementary Figure 3. Mating-type locus for sexual reproduction in *C. auris*. **(a)** Phylogenetic tree of *C. auris* isolates from Lockhart et al. 2016. Isolates are color code according the clades (I, II, III and IV) and mating type (*MTL***a** and *MTL*α). **(b)** The normalized depth read coverage of mapped positions for all sequenced *C. auris* isolates from Lockhart *et al*. (2016) mapped to B8441 (*MTL***a**) and B11221 (*MTL*α) were computed for each specific *MTL* linked gene -*OPB***a**, *PIK***a**, and *PAP***a**- and *OPB*α, *PIK*α, and *PAP*α-, respectively. Branch lengths indicate the mean number of changes per site.

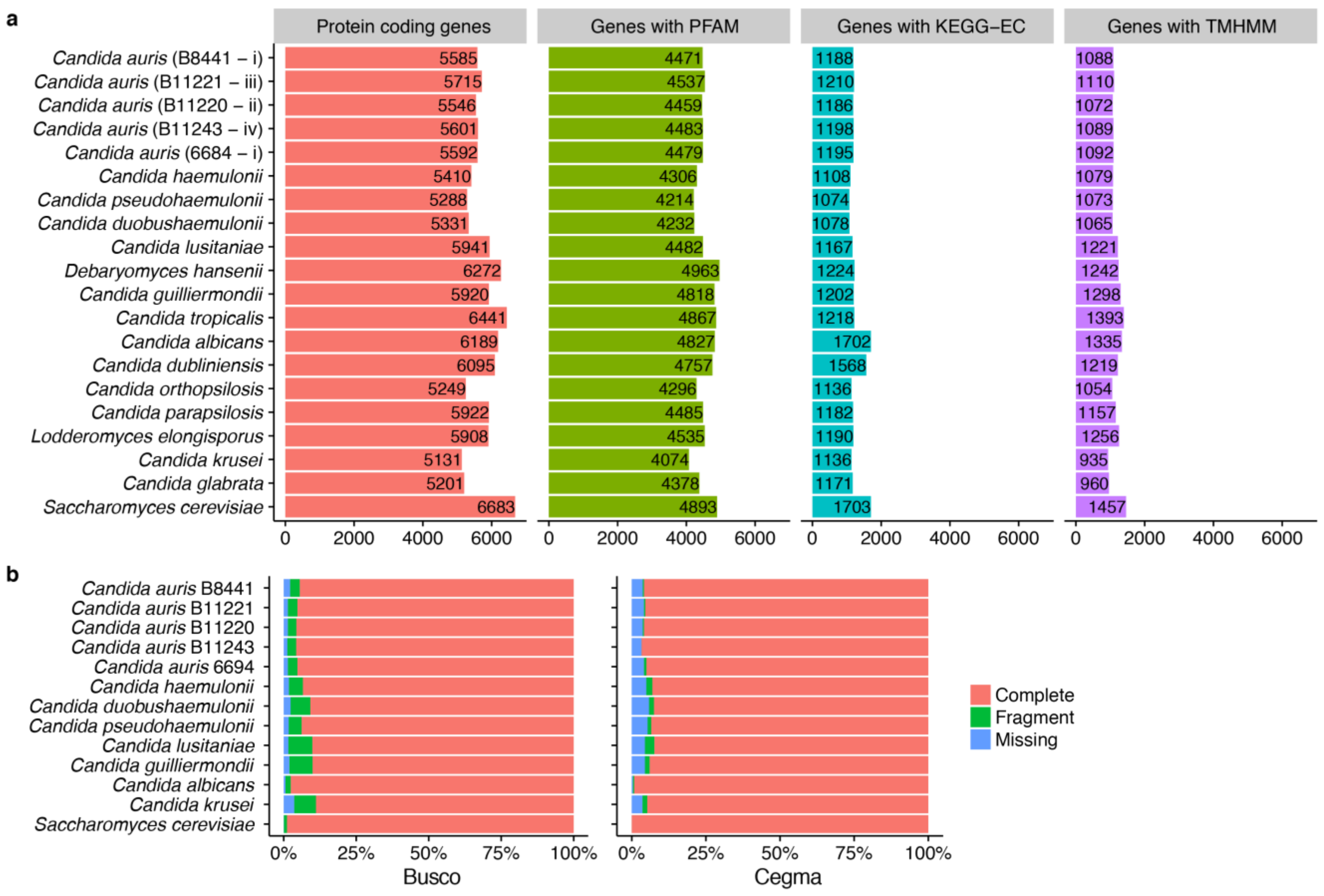

Supplementary Figure 4. Overall description and comparison of the annotated genome assemblies included in this study. **(a)** Bar plot depicts number of protein-coding genes, and functional annotation (protein family domain PFAM; pathways KEGG-EC; transmembrane domain TMHMM). **(b)** Conservation of core eukaryotic genes (CEGs) using BUSCO and CEGMA across the genomes of *Candida auris* strains B8441, B11221, 6694, B11220, B11243, *C. haemulonii* (B11899), *C. pseudohaemulonii* (B12108), *C. duobushaemulonii* (B09383), and other genomes of closely related Saccharomycetales species included in comparative genomic analyses. Roman numerals indicate the corresponding clade for *C. auris* isolates.

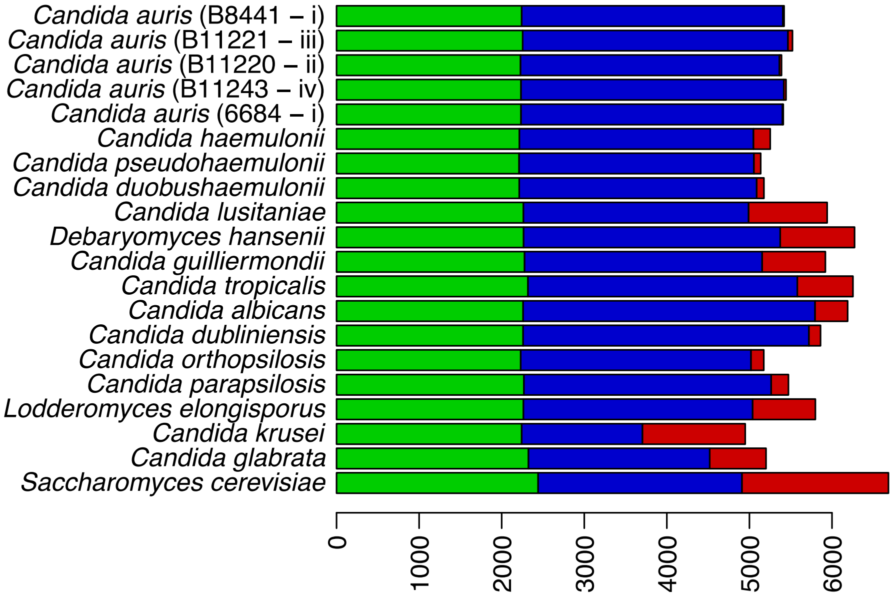

Supplementary Figure 5. Distribution of ortholog clusters between *Candida auris*, and closely related pathogenic and non-pathogenic species from the order Saccharomycetales. A bar plot of orthology classes is shown, where core genes found in all genomes are shown in green, shared genes present in more than one but not all genomes in blue, and genes that were unique to only one of the 20 genomes in red. Roman numerals indicate the corresponding clade for *C. auris* isolates.

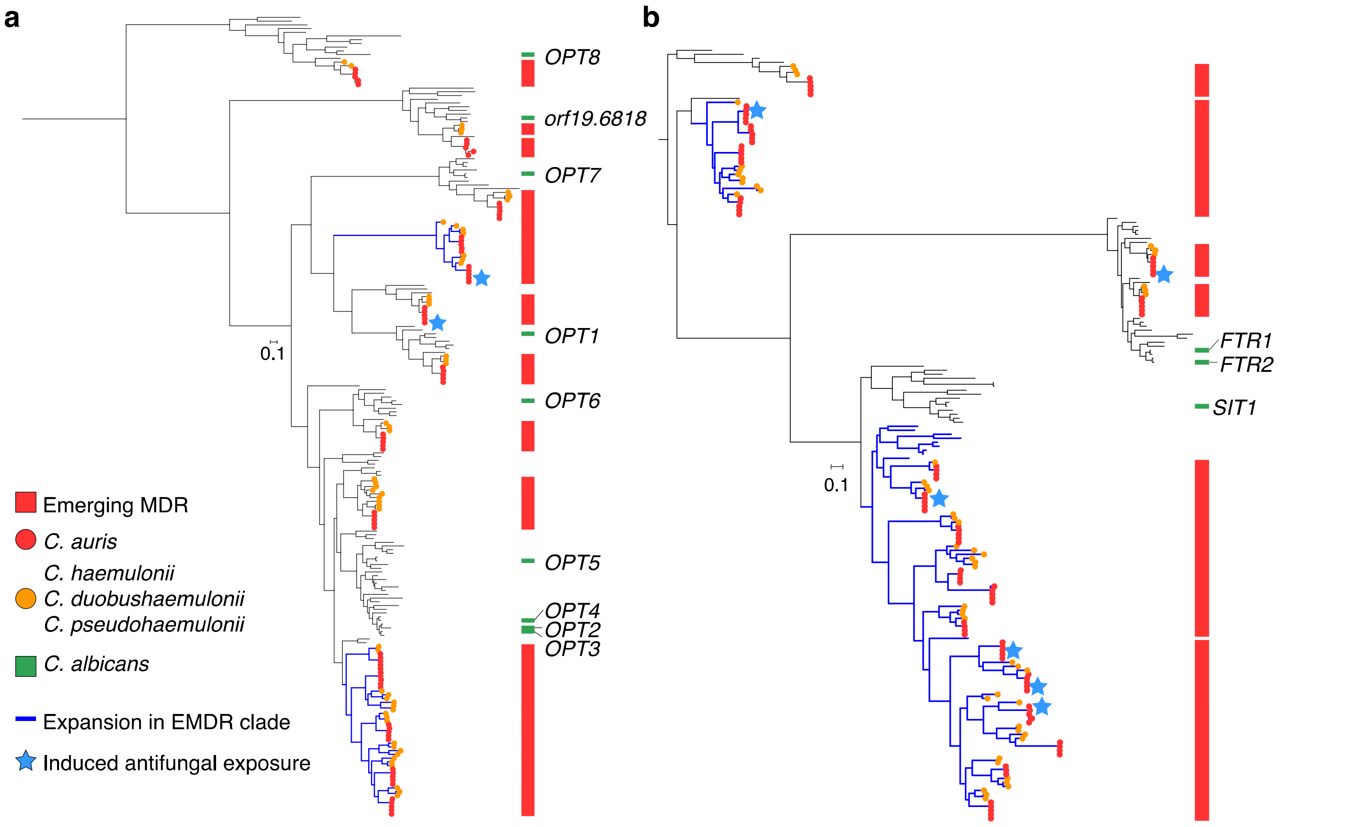

Supplementary Figure 6. Phylogenetic relationships of oligopeptide transporters (*OPT*) and siderophore iron transporters (*SIT*) families. Phylogenetic trees estimated by maximum likelihood with RAxML showing expansion of *OPT* **(a)** and *SIT* **(b)** transporter families in emerging multidrug-resistant (MDR) species *C. auris* and *C. haemulonii*, *C. duobushaemulonii* and *C. pseudohaemulonii*. Each species has a color code and lineage-specific expansions (blue branches) can be seen in *C. auris* and closely related species relative to the close ancestor *C. lusitaniae* and *C. albicans*. Orthologs of *OPT* and *SIT* transporters in *C. albicans* are depicted along side each tree. Branch lengths indicate the mean number of changes per site.

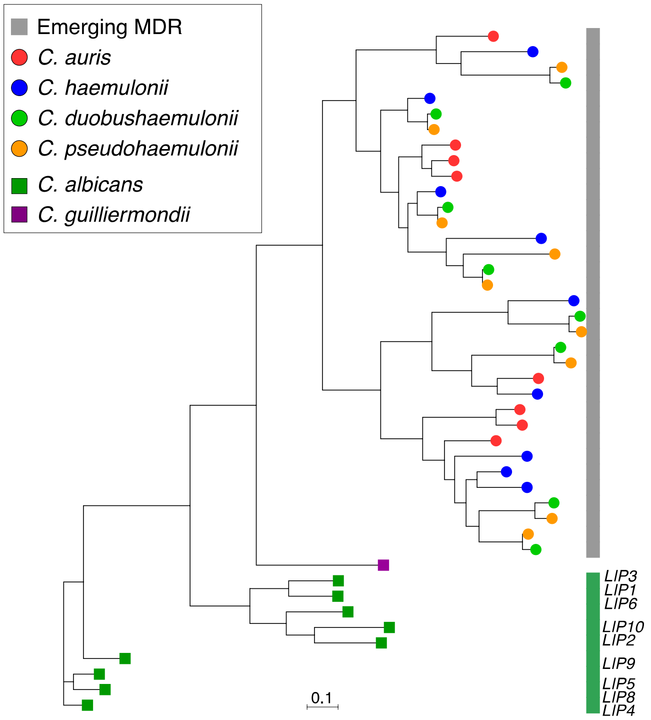

Supplementary Figure 7. Phylogeny of secreted lipases. Phylogenetic trees estimated by maximum likelihood with RAxML showing expansion of secreted lipases (*LIP*) in multidrug-resistant (MDR) species *C. auris* and *C. haemulonii*, *C. duobushaemulonii* and *C. pseudohaemulonii*. Each species has a color code. Orthologs of *LIP*(s) in *C. albicans* are depicted along side each tree. Branch lengths indicate the mean number of changes per site.

Amino acid substitution	Q21R	F105L	S110A	A114V	D116A	K119S	F126L	Y132F	K143R	F145L	D153E	T229A	Y257A	M258L	E266D	R267T	D278N	H283R	G307S	L403F	S405F	A432S	V437I	N440D	D446E	Y447S	G448E	F487Y	F449I	G450E	G464S	I471M	I483V	V488I
Candida albicans	Q	F	S	A	D	K	F	Y	K	F	D	T	Y	M	E	R	D	H	G	L	S	A	V	N	D	Y	G	F	F	G	G	I	I	V
C. auris (B8441 – I)	V	L	A	A	A	S	F	Y	K	F	E	T	Y	M	K	T	D	N	G	M	S	S	–	I	D	Y	G	Y	F	G	G	I	I	V
C. auris (6684 – I)	V	L	A	A	A	S	F	F	K	F	E	T	Y	M	K	T	D	N	G	M	S	S	–	I	D	Y	G	Y	F	G	G	I	I	V
C. auris (B11221 – III)	V	L	A	A	A	S	L	Y	K	F	E	T	Y	M	K	T	D	N	G	M	S	S	–	I	D	Y	G	Y	F	G	G	I	I	V
C. auris (B11220 – II)	V	L	A	A	A	S	F	Y	K	F	E	T	Y	M	K	T	D	N	G	M	S	S	–	I	D	Y	G	Y	F	G	G	I	I	V
C. auris (B11243 – IV)	V	L	A	A	A	S	F	F	K	F	E	T	Y	M	K	T	D	N	G	M	S	S	–	I	D	Y	G	Y	F	G	G	I	I	V
C. haemulonii	V	L	A	A	A	S	F	Y	K	F	E	T	Y	M	K	N	D	N	G	M	S	L	–	I	D	Y	G	Y	F	G	G	I	I	V
C. duobushaemulonii	V	L	A	A	A	A	F	Y	K	F	E	T	Y	M	K	T	D	N	G	M	S	S	–	I	D	Y	G	Y	F	G	G	I	I	V
C. pseudohaemulonii	V	L	A	A	A	S	F	F	K	F	E	T	Y	M	K	T	D	N	G	M	S	S	–	I	D	Y	G	Y	F	G	G	I	I	V
C. lusitaniae	S	L	A	A	E	S	F	Y	K	F	D	T	Y	M	K	S	D	N	G	L	S	A	–	S	D	Y	G	Y	F	G	G	I	I	V
C. krusei	Q	L	S	A	D	T	F	Y	K	F	E	T	Y	L	A	G	D	N	G	L	S	S	I	N	D	Y	G	Y	F	G	G	I	L	I
C. glabrata	Q	F	A	A	A	S	F	Y	K	F	E	T	Y	M	E	K	D	N	G	L	S	A	–	A	D	Y	G	F	F	G	G	I	L	I

Supplementary Figure 8. Multiple alignment of the azole drug target *ERG11.* Observed amino acid substitutions in *C. albicans* compared with *Candida auris* strains B8441, B11221, 6694, B11220, B11243, *C. haemulonii* (B11899), *C. pseudohaemulonii* (B12108) and *C. duobushaemulonii* (B09383). Red boxes indicate observed azole resistance amino acids substitutions in *C. auris* isolates and *C. haemulonii*, *C. pseudohaemulonii* and *C. duobushaemulonii*. Amino acid numbers are based on *C. albicans* protein sequence. Depicted amino acid substitutions in *ERG11* were observed in previous studies and reported in Lockhart et al., 2016 and Flowers et al., 2014.

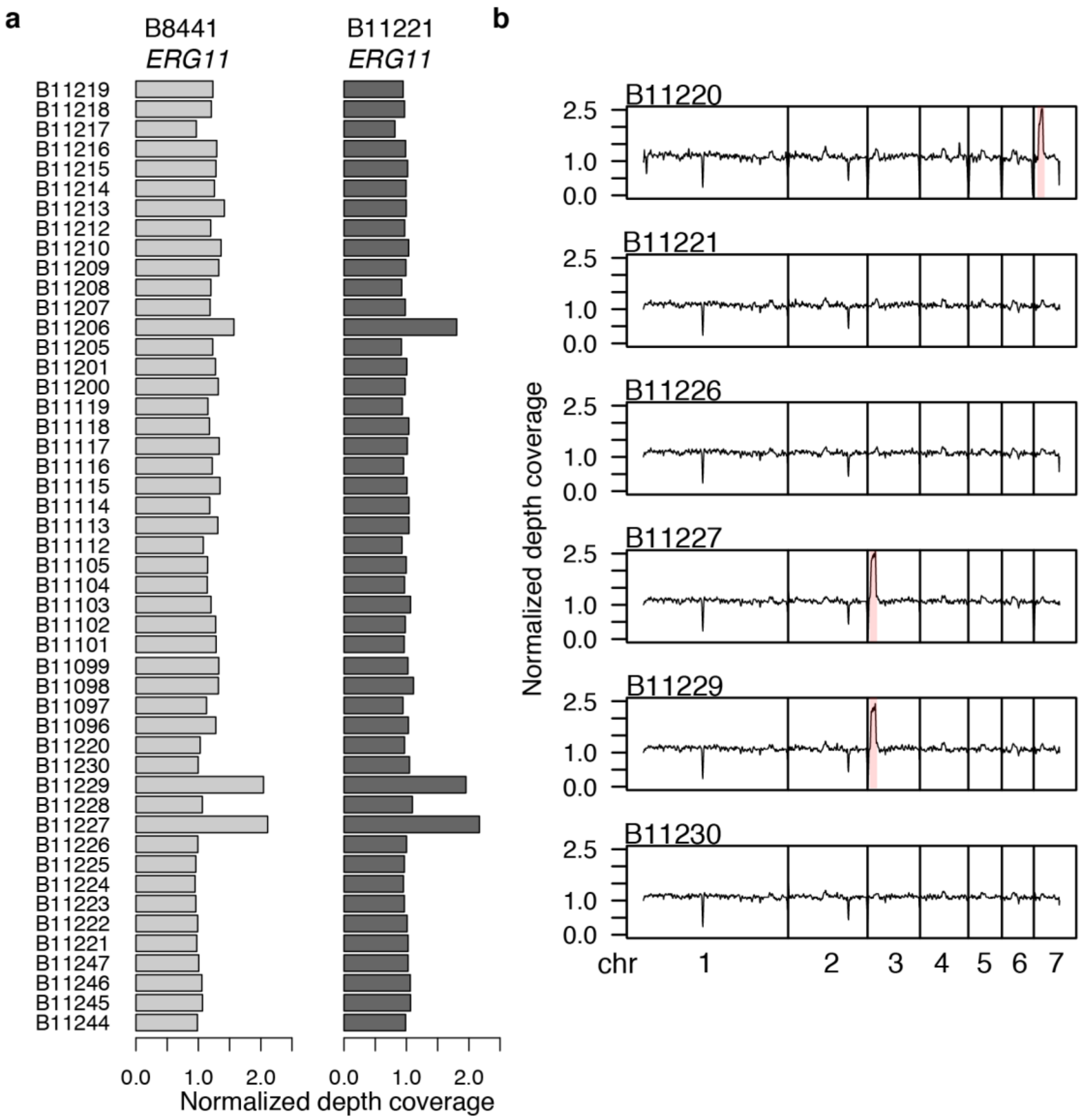

Supplementary Figure 9. Copy number variation (CNV) of *ERG11* in *C. auris*. **(a)** The normalized depth read coverage of mapped positions for sequenced *C. auris* isolates from Lockhart *et al*. (2016) mapped to B8441 (*MTL***a**) and B11221 (*MTL*α) were computed for *ERG11*. **(b)** Normalized read density distribution in 48 *C. auris* isolates. Each panel corresponds to one strain; the normalized sequence depth (*y-axis*) centered on 1X is shown across the genome assembly of *C. auris* B11221 reference (*x-axis*, scaffolds 1 to 7). Shaded red boxes highlight large duplicated regions or CNV tracts based on normalized read density.

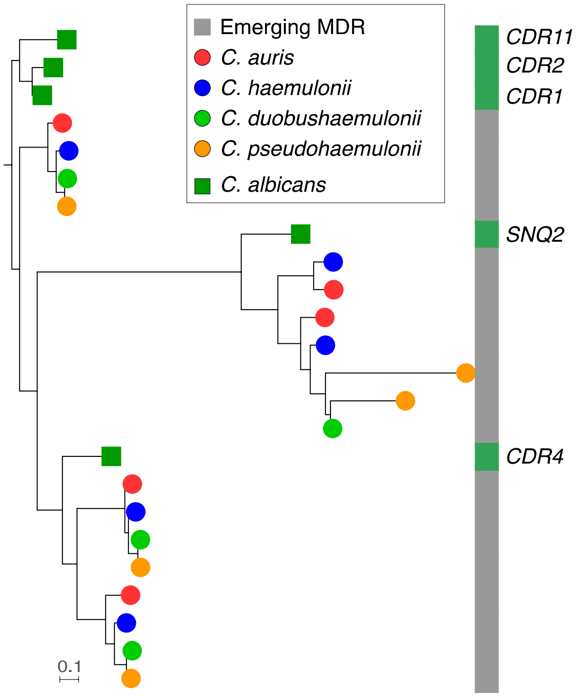

Supplementary Figure 10. Phylogeny of known multidrug transporters. Phylogenetic trees estimated by maximum likelihood with RAxML showing phylogenetic relationships of multidrug transporters in multidrug-resistant (MDR) species *C. auris* and *C. haemulonii*, *C. duobushaemulonii* and *C. pseudohaemulonii*. Each species has a color code. Orthologs of multidrug transporters in *C. albicans* are depicted along side each tree. Branch lengths indicate the mean number of changes per site.

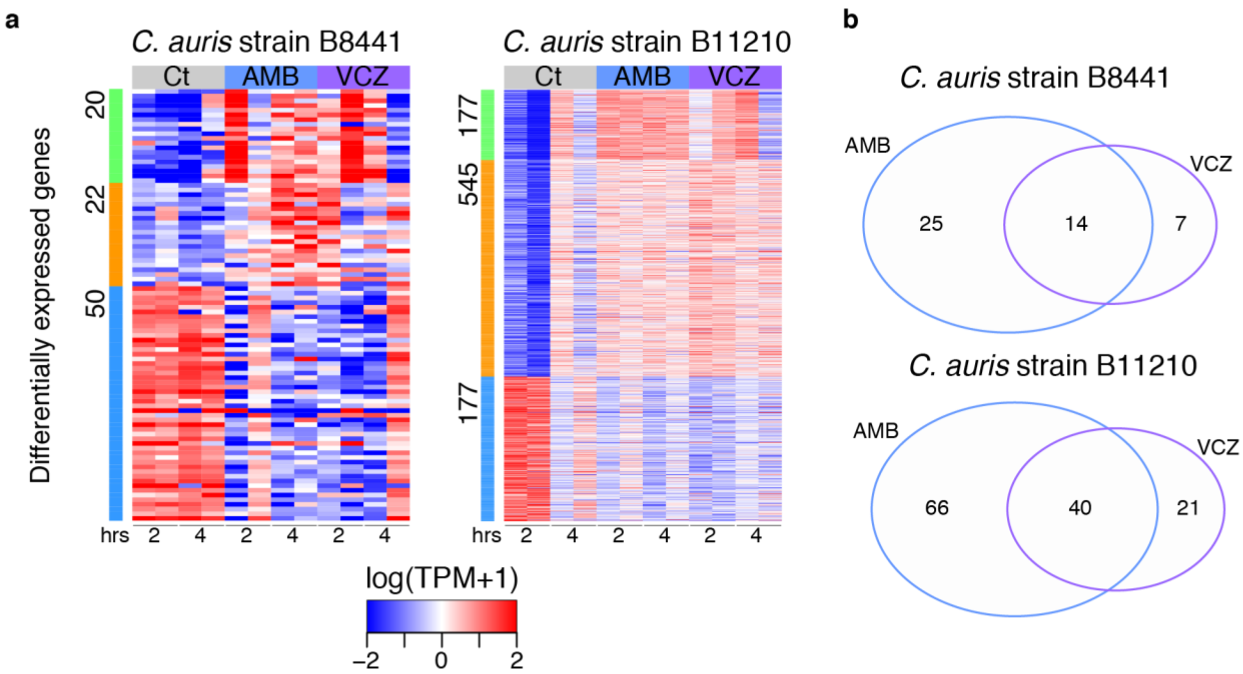

Supplementary Figure 11. Transcriptional response during antifungal treatment in *C. auris*. **(a)** Heatmap depicts significantly differentially expressed genes (DEGs; fold-change >2, false discovery rate < 0.05) for *C. auris* strain B8441 (left) and B11210 (right) at 2 and 4 hours during amphotericin B (AMB) and voriconazole (VCZ) treatment. DEGs were grouped by *k-means* (similar expression patterns) into three clusters. Green and orange clusters correspond to genes induced, and blue cluster corresponds to genes repressed during antifungal drug exposure. TPM: transcripts per million. **(b)** Venn diagram depicts number of induced genes in *C. auris* isolate B8441 (DEGs; FC >2, FDR < 0.05; Table S10 sheet1) and isolate B11210 (DEGs; FC >4, FDR < 0.001; Table S10 sheet2) during amphotericin B (AMB) and voriconazole (VCZ) treatment.

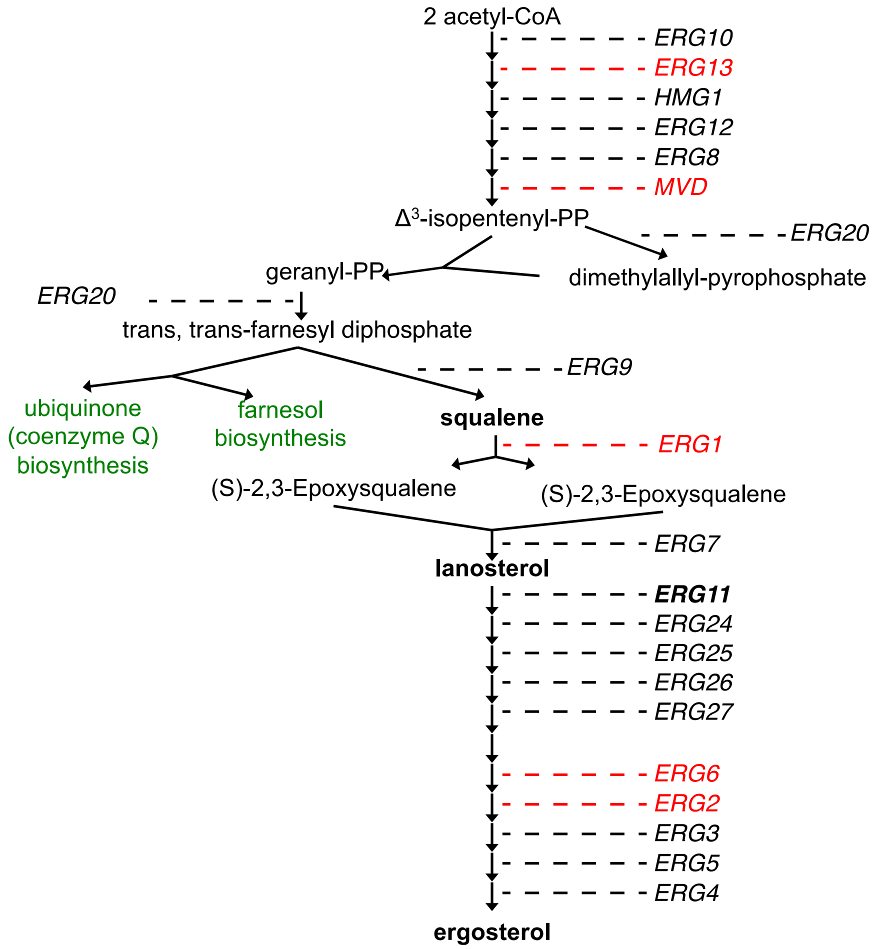

Supplementary Figure 12. Ergosterol biosynthesis pathway. Summarized ergosterol pathway in *Candida auris*. Genes in red were induced (fold-change >4, false discovery rate < 0.001) in *C. auris* resistant isolate B11210 (Amphotericin B MIC > 4) upon treatment with Amphotericin B. Table S6 includes the genes IDs for genes in the ergosterol pathway in *C. auris* and closely related species.

SUPPLEMENTARY TABLES

Supplementary Table 1. Antifungal susceptibility testing for isolates included in this study*

Species	Strain	Clade	Fl	Vcz	Amb	Cs	And	5fc
Candida auris	B8441	I	8	0.06	0.75	0.25	0.5	8
Candida auris	B11221	III	64	0.5	0.125	0.25	0.5	0.5
Candida auris	B11220	II	4	0.03	0.38	0.12	0.25	2
Candida auris	B11243	IV	>256	8	0.75	0.5	1	0.5
Candida auris	B11210	III	128	1	4	0.03	0.5	0.12
Candida auris	B11227	III	128	4	0.38	0.5	1	0.5
Candida auris	B11229	III	64	8	0.38	0.25	0.5	0.5
Candida auris	B11206	I	256	2	1.5	0.06	0.12	0.5
Candida duobushaemulonii	B09383	.	8	0.06	>32	0.016	0.015	.
Candida haemulonii	B11899	.	64	0.5	8	0.5	0.03	0.12
Candida pseudohaemulonii	B12108	.	256	1	>32	0.125	0.25	.

*Fluconazole (Fl); Voriconazole (Vcz); Amphotericin B (Amb); Caspofungin (Cs); Anidulafungin (And); Flucytosine (5fc).
Resistance breakpoints are approximated as follows. Fl >=32; Amb >=2; Cs >=2; And >=4.

Supplementary Table 2. Species included in this study

**Species**	**Strain**	**Clade**	**Accession ID**
Candida auris	B8441	I	PEKT00000000
Candida auris	B11221	III	PGLS00000000
Candida auris	B11220	II	PYFR00000000
Candida auris	B11243	IV	PYGM00000000
Candida haemulonii	B11899	.	PKFO00000000
Candida duobushaemulonii	B09383	.	PKFP00000000
Candida pseudohaemulonii	B12108	.	PYFQ00000000
Candida auris	6684	I	.
Candida lusitaniae	.	.	.
Debaryomyces hansenii	.	.	.
Candida guilliermondii	.	.	.
Candida tropicalis	.	.	.
Candida albicans	.	.	.
Candida dubliniensis	.	.	.
Candida orthopsilosis	.	.	.
Candida parapsilosis	.	.	.
Lodderomyces elongisporus	.	.	.
Candida krusei	.	.	.
Candida glabrata	.	.	.
Saccharomyces cerevisiae	.	.	.

bold: assembled and annotated genomes in this study

Supplementary Table 3. Conservation of mating type genes

Gene	Cau (B8441)	Cau (B11221)	Cau (B11220)	Cau (B11243)	*Cha*	*Cps*	*Cdu*
PAP1	B9J08_001453	CJI97_001149	CJI96_001795	CJJ07_004260	CXQ85_003096	C7M61_004932	CXQ87_003540
OBPA	B9J08_001454	CJI97_001151	CJI96_001793	CJJ07_004261	CXQ85_003094	C7M61_004931	CXQ87_003538
PIKA	B9J08_001455	CJI97_001150	CJI96_001794	CJJ07_004262	CXQ85_003095	C7M61_004930	CXQ87_003539
SLA2	B9J08_001427	CJI97_001177	CJI96_005494	CJJ07_002768	CXQ85_003069	C7M61_002716	CXQ87_003512
MAS2	B9J08_001452	CJI97_001152	CJI96_001791	CJJ07_004259	CXQ85_003093	C7M61_004933	CXQ87_003536
RCY1	B9J08_001457	CJI97_001147	CJI96_001797	CJJ07_004264	CXQ85_003099	C7M61_004928	CXQ87_003541
MTLa1	B9J08_005589	.	.	CJJ07_005605	.	C7M61_005294	.
MTLa2	B9J08_005590	.	.	CJJ07_005606	.	C7M61_005295	.
MTLalpha1	.	CJI97_005719	CJI96_005550	.	CXQ85_005415	.	CXQ87_005336

Cau = Candida auris; Cha = C. haemulonii; *Cdu = C. duobushaemulonii*; *Cps = C. pseudohaemulonii*

Supplementary Table 4. Number of differentially expressed* during drug treatment with amphotericin B or vorizonazole in *Candida auris*

	B11210-N-2hr	B11210-N-4hr	B11210-A-2hr	B11210-A-4hr	B11210-V-2hr	B11210-V-4hr	B8441-N-2hr	B8441-N-4hr	B8441-A-2hr	B8441-A-4hr	B8441-V-2hr	B8441-V-4hr
B11210-N-2hr												
B11210-N-4hr	0											
B11210-A-2hr	701(162)	0										
B11210-A-4hr	0	0 (0)	0									
B11210-V-2hr	945 (96)	0	37	0								
B11210-V-4hr	0	1 (0)	0	1	0							
B8441-N-2hr	1183 (352)	0	0	0	0	0						
B8441-N-4hr	0	0 (0)	0	0	0	0	0					
B8441-A-2hr	0	0	1	0	0	0	13 (5)	0				
B8441-A-4hr	0	0	0	10	0	0	0	69 (11)	0			
B8441-V-2hr	0	0	0	0	12	0	37 (5)	0	0	0		
B8441-V-4hr	0	0	0	0	0	0	0	2 (2)	0	2	0	

*Differentially expressed genes (Fold change >4, False Discovery rate < 0.001). Number of genes in parenthesis: Fold change >2, False Discovery rate < 0.05.
