# Supplementary material for: Genomic insights into multidrug-resistance, mating and virulence in Candida auris and related emerging species
Source: Nat Commun. 2018 Dec 17;9:5346. doi: 10.1038/s41467-018-07779-6 (PMC6297351; doi:10.1038/s41467-018-07779-6)
Supplement: Supplementary file 2 — Description of Additional Supplementary Files [file 41467_2018_7779_MOESM2_ESM.pdf]

Description of Additional Supplementary Files

File Name: Supplementary Data 1
Description: Conservation of meiosis genes in Candida auris and closely related species.

File Name: Supplementary Data 2
Description: Gene conservation analysis and protein family enrichment analysis in Candida auris and closely related species.

File Name: Supplementary Data 3
Description: Conservation of ergosterol biosynthesis pathway genes in Candida auris and closely related species.

File Name: Supplementary Data 4
Description: Duplicated regions and genes found in these regions in Candida auris isolates.

File Name: Supplementary Data 5
Description: Gene copy number variation and conservation in ERG11 and genes associated with drug resistance

File Name: Supplementary Data 6
Description: Genes differentially expressed (Fold change [FC] >4; False discovery rate [FDR] < 0.001) during drug treatment with amphotericin B or voriconazole in C. auris isolates B8441 and B11210.

File Name: Supplementary Data 7
Description: Genes differentially expressed (Fold change [FC] >4; False discovery rate [FDR] < 0.001) between untreated C. auris isolates B8441 and B11210.

File Name: Supplementary Data 8
Description: Genetic variants, effect and impact in Candida auris B8441 and B11210.
