# Supplementary material for: Genomic insights into multidrug-resistance, mating and virulence in Candida auris and related emerging species
Source: Nat Commun. 2018 Dec 17;9:5346. doi: 10.1038/s41467-018-07779-6 (PMC6297351; doi:10.1038/s41467-018-07779-6)
Supplement: Supplementary file 11 — Reporting Summary [file 41467_2018_7779_MOESM11_ESM.pdf]

nature research

Corresponding author(s): Christina Cuomo, Anastasia Litvintseva

Reporting Summary

Nature Research wishes to improve the reproducibility of the work that we publish. This form provides structure for consistency and transparency in reporting. For further information on Nature Research policies, see Authors & Referees and the Editorial Policy Checklist.

Statistical parameters

When statistical analyses are reported, confirm that the following items are present in the relevant location (e.g. figure legend, table legend, main text, or Methods section).

n/a	Confirmed	
☐	☒	The exact sample size (n) for each experimental group/condition, given as a discrete number and unit of measurement
☐	☒	An indication of whether measurements were taken from distinct samples or whether the same sample was measured repeatedly
☐	☒	The statistical test(s) used AND whether they are one- or two-sided<br>*Only common tests should be described solely by name; describe more complex techniques in the Methods section.*
☒	☐	A description of all covariates tested
☐	☒	A description of any assumptions or corrections, such as tests of normality and adjustment for multiple comparisons
☐	☒	A full description of the statistics including central tendency (e.g. means) or other basic estimates (e.g. regression coefficient) AND variation (e.g. standard deviation) or associated estimates of uncertainty (e.g. confidence intervals)
☐	☒	For null hypothesis testing, the test statistic (e.g. F, t, r) with confidence intervals, effect sizes, degrees of freedom and P value noted<br>*Give P values as exact values whenever suitable.*
☒	☐	For Bayesian analysis, information on the choice of priors and Markov chain Monte Carlo settings
☒	☐	For hierarchical and complex designs, identification of the appropriate level for tests and full reporting of outcomes
☐	☒	Estimates of effect sizes (e.g. Cohen's d, Pearson's r), indicating how they were calculated
☒	☐	Clearly defined error bars<br>*State explicitly what error bars represent (e.g. SD, SE, CI)*

Our web collection on statistics for biologists may be useful.

Software and code

Policy information about availability of computer code

Data collection

Genomic DNA was extracted using the Quick-DNA™ (ZR) Fungal/Bacterial Miniprep Kit (Zymo Research, Irvine, CA, USA)
Genomic libraries were constructed and barcoded using the NEBNext Ultra DNA Library Prep kit (New England Biolabs, Ipswich, MA, USA)
Genome sequencing with Illumina HiSeq 2500 (HiSeq Rapid SBS Kit v2) , Illumina MiSeq (MiSeq Reagent Kit v2), and PacBio (MasterPure™ Yeast DNA Purification Kit; PacBio RS II SMRT DNA sequencing system)
Optical maps were generated using the OpGen optical mapping platform (OpGen)

RNA was isolated using RiboPure™-Yeast rapid RNA isolation kit (Life technologies, Carlsbad, CA).
RNA was adapted for sequencing using the RNAtag-Seq approach, with the yeast RiboZero reagent used for rRNA depletion

Data analysis	Genome assembly: Canu v1.6, Circlator v1.5, Quiver, part of SmrtAnalysis suite v2.3, SPAdes v3.1.137, Pilon v1.16, GAEMR package (http://software.broadinstitute.org/software/gaemr/)<br><br>Gene annotation: Tophat2 v2.0.8, BRAKER1, GeneMark-ET, AUGUSTUS, HMMER3, Blast2GO, BLAST<br><br>Phylogenomics: OrthoMCL v1.4, MUSCLE v3.8.31, RAxML v7.7.8, iTOL (https://itol.embl.de/login.cgi)<br><br>Transcriptional analysis: Bowtie2 v.1.2.21, Rsem v1.2.21, edgeR v3.10.5 Trinity v2.1.1, R Studio v1.0.143

For manuscripts utilizing custom algorithms or software that are central to the research but not yet described in published literature, software must be made available to editors/reviewers upon request. We strongly encourage code deposition in a community repository (e.g. GitHub). See the Nature Research guidelines for submitting code & software for further information.

Data

Policy information about availability of data

All manuscripts must include a data availability statement. This statement should provide the following information, where applicable:

- Accession codes, unique identifiers, or web links for publicly available datasets
- A list of figures that have associated raw data
- A description of any restrictions on data availability

All genome assemblies and gene annotations have been deposited at DDBJ/EMBL/GenBank under the following accession numbers: C. auris B8441 PEKT00000000; C. auris B11221 PGLS00000000, C. auris B11220 PYFR00000000, C. auris B11243 PYGM00000000, C. haemulonii B11899 PKFO00000000, C. duobushaemulonii B09383 PKFP00000000, C. pseudohaemulonii B12108 PYFQ00000000. The RNA-Seq data from C. auris has been deposited at GenBank under Bioproject PRJNA445471.

Field-specific reporting

Please select the best fit for your research. If you are not sure, read the appropriate sections before making your selection.

☒ Life sciences ☐ Behavioural & social sciences ☐ Ecological, evolutionary & environmental sciences

For a reference copy of the document with all sections, see nature.com/authors/policies/ReportingSummary-flat.pdf

Life sciences study design

All studies must disclose on these points even when the disclosure is negative.

Sample size	Seven whole genome sequences were fully assembled and annotated. Four for Candida auris and one each for C. haemulonii, C. duobushaemulonii, C. pseudohaemulonii.<br><br>Two C. auris strains were used for RNA-Seq and transcriptional analysis during drug treatment.<br><br>Fifty C. auris isolates previously sequenced, encompassing 4 included in this study, were used for population genomics analysis.
Data exclusions	No data was excluded
Replication	For RNA-Seq analysis we performed two biological replicates for each of the conditions
Randomization	For protein family expansion analysis we determined two groups 1) Emerging multidrug-resistant species and 2) Other species from the order Saccharomycetales
Blinding	We were blinded during data collection and analysis

Reporting for specific materials, systems and methods

Materials & experimental systems

n/a	Involved in the study
☐	☒ Unique biological materials
☒	☐ Antibodies
☒	☐ Eukaryotic cell lines
☒	☐ Palaeontology
☒	☐ Animals and other organisms
☒	☐ Human research participants

Methods

n/a	Involved in the study
☒	☐ ChIP-seq
☒	☐ Flow cytometry
☒	☐ MRI-based neuroimaging

Unique biological materials

Policy information about availability of materials

Obtaining unique materials	All the strains sequenced and analyzed in this study are available at the Center for Disease Control and Prevention (CDC). In addition, all raw sequenced data from whole genome sequencing and RNA-Seq data had been deposited in GenBank.
